# Protective Effects of Rhamnetin in Carbapenem-Resistant *Acinetobacter baumannii*-Induced Sepsis Model and the Underlying Mechanism

**DOI:** 10.3390/ijms242115603

**Published:** 2023-10-26

**Authors:** Minju Kim, Shubhash Chandra Chaudhary, Byeongkwon Kim, Yangmee Kim

**Affiliations:** Department of Bioscience and Biotechnology, Konkuk University, Seoul 05029, Republic of Korea; alswn7074@konkuk.ac.kr (M.K.); schandrachaudhary@gmail.com (S.C.C.); matt97@konkuk.ac.kr (B.K.)

**Keywords:** antioxidant, apoptosis, carbapenem-resistant *Acinetobacter baumannii*, rhamnetin, ROS, sepsis

## Abstract

Carbapenem-resistant *Acinetobacter baumannii* (CRAB) is a well-known harmful bacterium that causes severe health disorders and dysregulates the host immune response associated with inflammation. Upon examining the suppressive activity of natural flavonoid rhamnetin on various pro-inflammatory cytokines in a CRAB-induced septic shock mouse model, we found that rhamnetin inhibited the production of IL-1β and IL-18, two pro-inflammatory cytokines associated with pyroptotic cell death, a process dependent on caspase-1. In this study, we investigated the antioxidant and anti-apoptotic activities of rhamnetin and the underlying mechanism of action in a CRAB infection. In the CRAB-induced septic shock mouse model, rhamnetin reduced the level of lipopolysaccharide (LPS) in lung lysates, resulting in the inhibition of TLR4-mediated inflammatory signaling. Notably, rhamnetin reduced intracellular reactive oxygen species (ROS) generation in macrophages and inhibited apoptotic and pyroptotic cell injury induced by CRAB infection. Therefore, rhamnetin inhibited LPS-induced pro-inflammatory mediators, hindering apoptotic and pyroptotic processes and contributing to a recovery effect in CRAB-induced sepsis mice by suppressing oxidative stress. Taken together, our study presents the potential role of rhamnetin in protecting against oxidative damage induced by CRAB infection through a TLR4 and ROS-mediated pyroptotic pathway, showing an alternative mechanism for sepsis prevention. Therefore, rhamnetin is a promising therapeutic candidate for treating CRAB-induced sepsis.

## 1. Introduction

The emergence of multidrug-resistant (MDR) Gram-negative bacteria is threatening global public health, and their infection causes serious sepsis, which is the leading cause of death worldwide [1]. Carbapenem antibiotics are effective in treating Gram-negative bacterial infections. However, excessive drug use increases bacterial resistance to carbapenem [2], especially carbapenem-resistant *Acinetobacter baumannii* (CRAB), which belongs to a group of critical pathogens requiring the development of new antibiotics, as urgently declared by the World Health Organization (WHO) [3]. Many researchers have attempted to develop new antibiotics to treat CRAB infections, particularly sepsis.

Gram-negative bacteria, such as CRAB, release various virulence factors, including lipopolysaccharide (LPS), an endotoxin, from the outer membrane [4]. LPS triggers a Toll-like receptor 4 (TLR4) signaling cascade [4] and causes a systemic inflammatory response, leading to organ dysfunction or sepsis due to uncontrolled inflammatory signaling during infection [5]. Sepsis is a broad term that has been defined in various ways up till now. It commonly refers to a life-threatening organ dysfunction caused by an unregulated response of a host to infection [6]. The early identification and timely care of patients have a great impact on reducing the mortality rate of the patients [7]. Owing to the failure of the initial lines of treatment, the mortality rate due to sepsis is in increasing order.

Currently, treatment options for CRAB infections are limited. Carbapenems, such as doripenem, imipenem, and meropenem, are generally considered the final choice of treatment for MDR Gram-negative bacteria [8]. However, many Gram-negative bacteria have adapted and developed resistance to carbapenem, making them difficult to treat and requiring the use of last-resort antibiotics, such as polymyxin B or colistin (polymyxin E) [9]. Nevertheless, owing to their potential toxicities, such as nephrotoxicity and neurotoxicity, the use of polymyxins has been limited. Because the mortality rate of sepsis caused by CRAB infection is very high, the development of new types of antibiotics is critical [10].

Reactive oxygen species (ROS) are short-lived molecules, such as hydrogen peroxide (H_2_O_2_), hydroxyl radical (OH^−^), superoxide (O_2_^−^), singlet oxygen (^1^O_2_), and nitric oxide (NO), which play a critical role in the regulation of inflammatory processes, leading to the oxidation of lipids and proteins [11]. In addition, ROS act as important signaling molecules that are crucial for host defense mechanisms, biosynthesis, and homeostasis processes, including immunity and metabolism, as well as for organismal physiology [12,13]. Nonetheless, excessive ROS generation by pathogens leads to oxidative stress in the host and is associated with various inflammatory diseases [14,15]. Uncontrolled acute inflammation caused by oxidative stress due to Gram-negative sepsis can result in cellular damage [16]. In LPS-stimulated macrophages, the production of ROS is upregulated, which causes inflammation and overproduction of inflammatory factors, further directly stimulating the generation of ROS [17]. However, LPS-induced ROS production in macrophages depends on the expression and activation of oxidative enzymes, such as Nicotinamide adenine dinucleotide phosphate (NADPH) oxidase, and the suppression of antioxidative enzymes, such as catalase and superoxide dismutase [11].

Pyroptosis is an inflammatory cell death characterized by cell swelling, disruption of cell membranes, and DNA cleavage [18]. Pyroptosis is primarily triggered by the activation of inflammasomes, followed by the cleavage of Gasdermin D and the formation of plasma membrane pores. More importantly, in pyroptosis, caspase-1 is activated and cleaved, which is accompanied by the release of pro-inflammatory intracellular components, such as IL-1β and IL-18, through cellular pores [19,20]. Numerous cellular stimuli, spanning from microbial pathogen-associated molecular patterns (PAMPs), such as LPS, to endogenous damage-associated molecular patterns (DAMPs), such as ATP and heat shock proteins, trigger the initiation of pyroptosis. This process involves the assembly of various inflammasome complexes, including those from the NOD-like receptor family and pyrin domain-containing protein 3 (NLRP3). These complexes recruit an apoptosis-associated speck-like protein containing a CARD (ASC), ultimately leading to the activation of caspase-1. This activation, in turn, facilitates the conversion of pro-caspase-1 into its active form [21,22]. The activated caspase-1 promotes the cleavage and subsequent release of pro-inflammatory cytokines to the extracellular space, triggering a form of inflammatory cell death known as pyroptosis [21]. Pyroptosis mainly occurs in macrophages and dendritic cells [19] and is associated with the release of pro-inflammatory cytokines, such as IL-1β and IL-18 [23,24].

**Figure 1:**
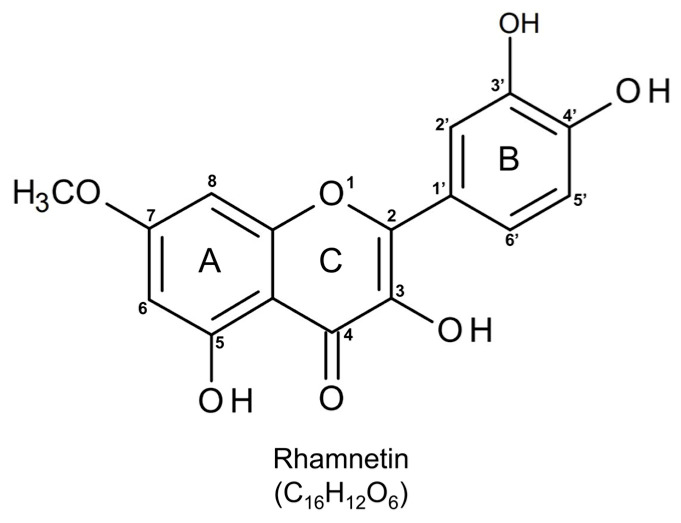
Chemical structure of rhamnetin (2-(3,4-dihydroxyphenyl)-3,5-dihydroxy-7-methoxychromen-4-one). The image was drawn using BIOVIA Draw software (version 17.1).

Flavonoids, which are secondary plant metabolites with a polyphenol-flavan skeleton, have extensive biological activities [25,26], and their antioxidant, anti-inflammatory, antibacterial, and antiviral effects make them attractive candidates for the treatment of bacterial infections [27,28]. We searched for naturally occurring flavonoids that could inhibit TLR4-mediated inflammatory signaling during Gram-negative infections [29]. We identified the anti-inflammatory activities of various flavonols, such as isorhamnetin, rhamnetin, and tamarixetin, which inhibit the production of pro-inflammatory cytokines in LPS-stimulated macrophages. [30,31,32]. In particular, rhamnetin, an O-methylated flavonol, can reduce inflammatory responses in the *E. coli* and CRAB-induced septic shock mouse model [31] (Figure 1). However, the underlying mechanism for the anti-inflammatory and antiseptic effects of rhamnetin has not been elucidated yet.

Rhamnetin is a flavonoid derived from *Rhamnus petiolaris*, *Syzygium aromaticum*, *Coriandrum sativum*, and *Prunus cerasus* [33]. Rhamnetin is O-methylated at position 7 of ring A and exerts various biological effects, including anti-inflammatory [34], anticancer [35,36], and antiseptic activities [31]. We previously observed that rhamnetin showed recovery effects on organ dysfunction and cytokine release in a CRAB-induced sepsis mouse model. Although rhamnetin did not possess strong antibacterial activity in vitro against Gram-negative bacteria, it showed potent antiseptic activity in a Gram-negative sepsis mouse model [31]. In this study, to explore the mechanism underlying its antiseptic activity in detail, we examined its suppressive activity on various pro-inflammatory cytokines in a CRAB-induced septic shock mouse model. We found that rhamnetin inhibited not only the production of TNF-α and IL-6 but also that of IL-1β and IL-18, which are known as the pro-inflammatory cytokines associated with caspase-1-dependent pyroptotic cell death. We found that rhamnetin robustly reduced CRAB-stimulated ROS generation, thereby inhibiting the cleavage of caspase-3, PARP-1, and caspase-1, indicating a cytoprotective role of rhamnetin against CRAB-mediated cell death. We elucidated that rhamnetin prevented oxidative damage in CRAB-induced sepsis in mice models and CRAB-induced murine macrophages by suppressing the ROS-mediated apoptotic and pyroptotic pathways. Therefore, to the best of our knowledge, our finding is a novel and alternative mechanism of rhamnetin’s protecting role against CRAB-induced sepsis. This study provides the potential therapeutic benefits of rhamnetin, a natural flavonoid, in the context of sepsis caused by CRAB infection and its underlying mechanism.

## 2. Results

### 2.1. Rhamnetin Has Suppressive Effects on IL-1β and IL-18 Production in CRAB-Induced Sepsis Mouse Model

**Figure 2:**
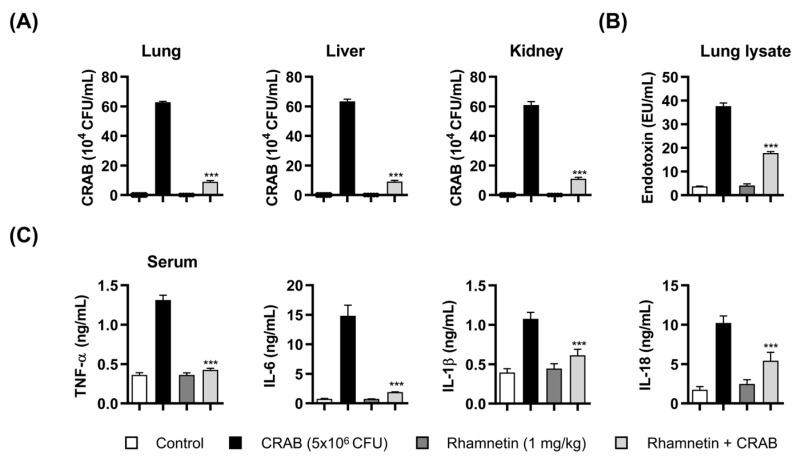
Antiseptic effects of rhamnetin in CRAB (5 × 10^6^ colony forming unit (CFU))-induced sepsis mouse model. (**A**) Bacterial counts (CFUs) in the lungs, liver, and kidneys. Suppressive effect of rhamnetin on (**B**) CRAB endotoxin levels measured using LAL assay in mice lung lysates and (**C**) cytokine levels (TNF-α, IL-6, IL-1β, and IL-18) in the serum. Data are presented as mean ± SEM. *** *p* < 0.001 compared with the CRAB-injected group.

To elucidate the antiseptic mechanism of rhamnetin in a CRAB-induced septic shock mouse model, we investigated its antiseptic effect by suppressing pyroptosis using a mouse model of CRAB-induced septic shock. The bacterial load in organs (lung, liver, and kidney) increased in the CRAB-induced sepsis mouse model, whereas treatment with rhamnetin (1 mg/kg) significantly reduced the bacterial levels in the lung, liver, and kidney by 87%, 85%, and 82%, respectively, compared to the CRAB-infected group (Figure 2A). We performed a Limulus amebocyte lysate (LAL) assay to examine the effect of rhamnetin on the level of LPS in mouse lung lysates in a CRAB-infected sepsis model. As shown in Figure 2B, the level of LPS in the CRAB-induced sepsis model treated with rhamnetin was markedly reduced (53%) compared to that in the CRAB-induced sepsis group. These results suggest that rhamnetin has a reducing effect on the LPS level in the CRAB sepsis mouse model, which plays an important role in the TLR4 signaling cascade. We analyzed the anti-inflammatory activity of rhamnetin in vivo (Figure 2C). Rhamnetin suppressed the serum levels of inflammatory cytokines, such as TNF-α and IL-6, by 68% and 87%, respectively. Furthermore, for the first time, we observed that rhamnetin suppressed the serum level of IL-1β and IL-18 by 43% and 47%, respectively. Pyroptosis is a caspase-1-dependent inflammatory cell death induced by the activation of inflammasomes that further activate caspase-1 and Gasdermin D. Consequently, pro-inflammatory cytokines, such as IL-1β and IL-18, are released into the extracellular space, ultimately leading to pyroptotic cell death. The results showed that pyroptosis mediated by CRAB infection in mice was extensively inhibited by rhamnetin. Therefore, in this study, we further investigated the protective role of rhamnetin against CRAB-induced sepsis.

### 2.2. Rhamnetin Exhibits Anti-Inflammatory Activities via TLR4 Signaling Pathway

**Figure 3:**
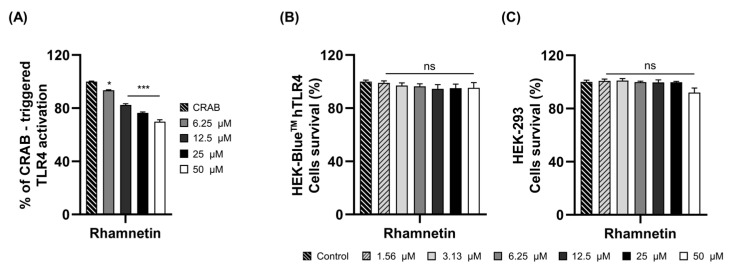
(**A**) Suppression of SEAP activity in CRAB-stimulated HEK-Blue™-hTLR4 cells with rhamnetin. Cytotoxicity of rhamnetin in (**B**) HEK-Blue™ hTLR4 cells and (**C**) HEK-293 cells. Results are shown as the mean ± SEM. * *p* < 0.05 and *** *p* < 0.001 compared with the CRAB group (**A**); ns, nonsignificant compared with control group (**B**,**C**).

We investigated the mechanisms underlying the anti-inflammatory activity of rhamnetin following CRAB stimulation. We first evaluated the inhibition of the TLR4-mediated inflammatory signaling pathway with rhamnetin in CRAB-induced inflammation. We performed a secreted embryonic alkaline phosphatase (SEAP) assay using HEK-Blue™ hTLR4 cells to confirm the inhibition of TLR4 inflammatory signaling with rhamnetin upon CRAB stimulation (Figure 3A). Results revealed that rhamnetin could reduce SEAP activity with a 24% and 30% inhibition rate at 25 μM and 50 μM, respectively, indicating its inhibitory effects toward the TLR4-mediated inflammatory signaling pathway in CRAB stimulation. To examine whether this TLR4 inhibitory activity was due to the toxicity of rhamnetin, we measured its toxicity in both HEK-Blue™ hTLR4 and HEK-293 cells at high concentrations up to 50 μM (Figure 3B,C). Rhamnetin showed cell viability over 95% and 92% at a concentration of 50 μM in HEK-Blue™ hTLR4 and HEK-293, respectively. These results imply that rhamnetin has the ability to inhibit TLR4-mediated signaling moderately without toxicity, even at a concentration of 50 μM.

### 2.3. Molecular Interactions between Rhamnetin and MD-2

**Figure 4:**
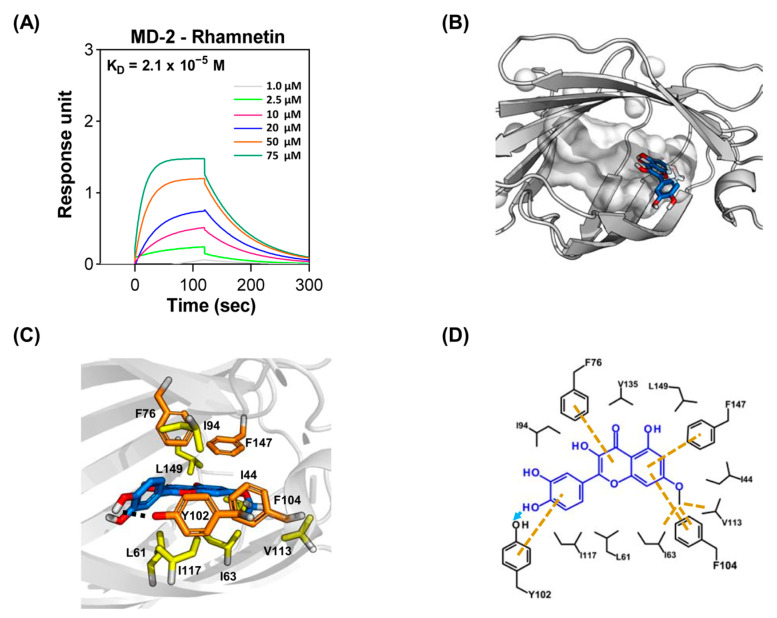
Binding interactions between MD-2 and rhamnetin. (**A**) Surface plasmon resonance (SPR) sensorgram of MD-2 interacting with rhamnetin. (**B**) Overview of the MD-2-rhamnetin complex. The hydrophobic cavity of MD-2 is depicted by the gray surface, and rhamnetin is depicted by the blue stick with red stick for oxygen atoms. (**C**) Overall complex of the MD-2–rhamnetin. Rhamnetin is depicted by the blue stick with red stick for oxygen atoms, hydrophobic interaction residues are shown as yellow sticks, and important aromatic residues are depicted by orange sticks in MD-2 (gray ribbon cartoon). (**D**) Two-dimensional diagram of the MD-2–rhamnetin docking pose showing hydrophobic interactions (yellow dash lines) and hydrogen bonding interaction (sky blue arrow).

We investigated the molecular interactions between rhamnetin and its receptors in the TLR4 signaling pathway. LPS released from Gram-negative bacteria is attracted by the LPS-binding protein and transferred to TLR4 via the adaptor protein MD-2, resulting in dimerization of the TLR4/MD-2 complex. The dimer mediates the translocation of nuclear factor-kappa B (NF-κB), ultimately leading to the production of pro-inflammatory cytokines. Since rhamnetin showed TLR4 inhibitory activity in the SEAP assay, we suspected that rhamnetin may inhibit the binding of LPS to the TLR4/MD-2 complex via binding to MD-2. Therefore, we measured the binding affinity of rhamnetin for MD-2 using surface plasmon resonance (SPR) and investigated its molecular interactions with MD-2. As shown in Figure 4A, the binding affinity of rhamnetin to MD-2 was 2.1 × 10^−5^ M (association rate: 6.9 × 10^2^ M^−1^ s^−1^; dissociation rate: 1.4 × 10^−2^ s^−1^). To understand the detailed intermolecular interactions between rhamnetin and MD-2, a binding model of rhamnetin to MD-2 was determined with a molecular docking simulation using the YASARA software (version 21.12.19) (Figure 4B–D). The binding model between rhamnetin and MD-2, with a binding energy of −7.5 kcal/mol, showed a strong hydrophobic interaction with the hydrophobic pocket (shown as a gray surface) of MD-2. As shown in Figure 4C, the 7-OCH3 of rhamnetin in ring A bound to the deep hydrophobic pocket of MD-2. In the case of the 7-OCH3 in ring A of rhamnetin, it formed hydrophobic interactions with Ile63, Phe104, and Val113. In addition, the A ring formed pi–pi interactions with Phe104 and Phe147. The B and C ring also formed pi–pi interactions with Tyr102 and Phe76, respectively. Furthermore, hydrophobic interactions were observed between the C ring of rhamnetin and Leu61 and Ile63, with one between the B ring and Ile94 and Ile117, as shown in the two-dimensional plot of Figure 4D. Therefore, rhamnetin may exert anti-inflammatory effects on CRAB-induced sepsis by inhibiting TLR4 activity through a direct interaction with MD-2. Despite the moderate inhibition of the TLR4 activity of rhamnetin, as shown in the SEAP assay, rhamnetin showed substantial antiseptic activity in a mouse model of sepsis, which led us to investigate further mechanisms by which it prevented sepsis.

### 2.4. Rhamnetin Inhibits ROS Generation in CRAB-Stimulated Murine Macrophages

**Figure 5:**
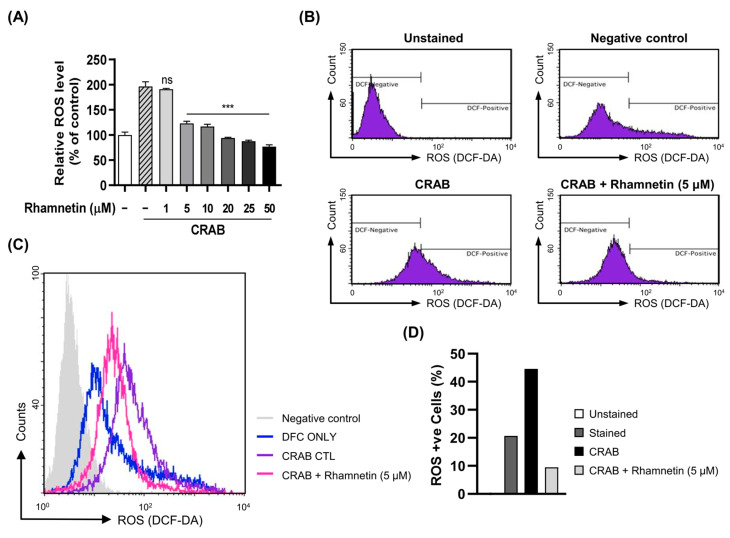
Rhamnetin inhibits ROS generation in CRAB (1 × 10^5^ CFU)-stimulated murine macrophages. (**A**) Inhibitory role of rhamnetin on CRAB-induced ROS production in murine macrophage cells treated with indicated doses of rhamnetin and stimulated with CRAB. (**B**) Flow cytometry analysis of intracellular ROS levels and the percentage of ROS-positive cells were assessed using flow cytometry analysis. (**C**,**D**) are histogram and bar graph, respectively, depicting ROS-positive cells in percentage. Results are shown as the mean ± SEM. *** *p* < 0.001 and ns, nonsignificant compared with the CRAB-stimulated group.

The excessive secretion of ROS disrupts various physiological and metabolic processes that ultimately lead to various human diseases. These include inflammatory diseases, tissue and organ damage, asthma, and even cancer [37,38]. Various reports have shown that LPS, an endotoxin produced by Gram-negative bacteria, exerts severe oxidative stress on macrophages owing to excessive ROS production, which activates the inflammatory signaling cascade. Recently, it was discovered that MDR *A. baumannii* invasion causes lung epithelial cell injury owing to oxidative stress triggered by ROS generation [39]. Therefore, we suspected that the macrophage cell damage caused by CRAB infection may be due to ROS production. Thus, we attempted to determine whether rhamnetin plays a crucial role in reducing intracellular ROS generation, which might protect cells from CRAB-induced cell death. We found that CRAB stimulated the production of ROS about two-fold higher than noninfected cells, and rhamnetin robustly attenuated CRAB-induced ROS generation in macrophages in a dose-dependent manner (Figure 5A). Furthermore, the population of ROS-positive cells, as analyzed using flow cytometry, dramatically increased to 45% with CRAB stimulation compared to nonstimulated cells. However, this increase in ROS-positive cells induced by CRAB stimulation was reduced to 9% following treatment with 5 μM rhamnetin (Figure 5B–D). These results provide further evidence that rhamnetin effectively inhibits CRAB-induced ROS production.

### 2.5. Rhamnetin Inhibits Apoptosis in LPS- and CRAB-Stimulated Murine Macrophages

**Figure 6:**
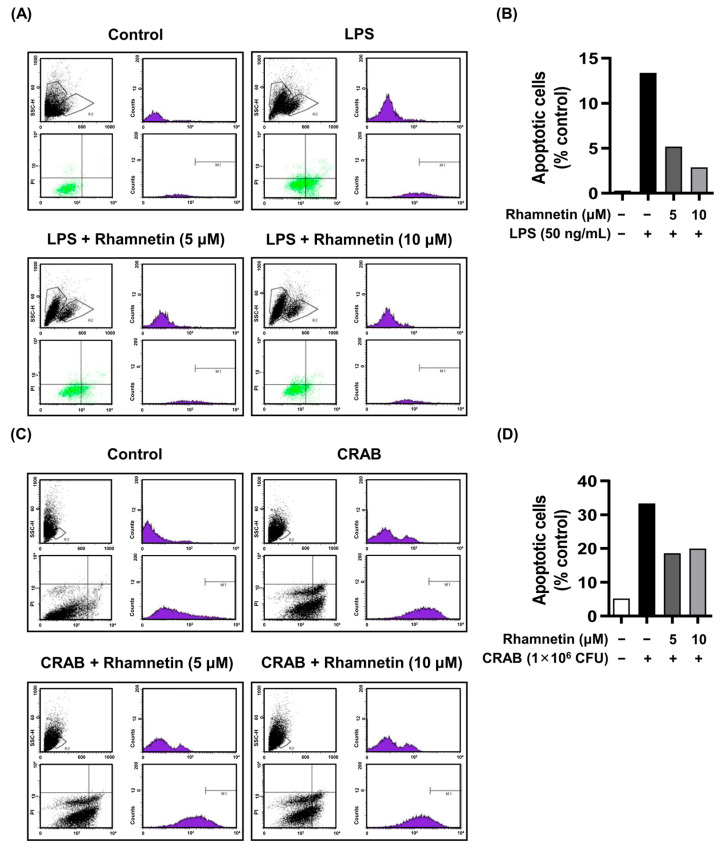
Rhamnetin inhibits apoptosis in LPS- and CRAB-stimulated murine macrophages. Flow cytometry analysis showing the inhibition of (**A**) LPS (50 ng/mL)- and (**C**) CRAB-induced apoptosis by indicated doses of rhamnetin. Bar graph depicting the number of apoptotic cells stimulated with (**B**) LPS and (**D**) CRAB.

Flow cytometry was used to confirm the cytoprotective role of rhamnetin against LPS- and CRAB-induced apoptosis. We found that apoptotic cell death dramatically increased to 13% upon LPS stimulation compared to that in nonstimulated cells (0.3%). The apoptotic cell death was intensely reduced to 5% and 3% with the treatment of rhamnetin with 5 μM and 10 μM, respectively (Figure 6A,B). The results indicate that rhamnetin plays a crucial role in cytoprotection against LPS-stimulated cell death.

Furthermore, CRAB robustly induced apoptosis approximately 7-fold higher (33%) than in nontreated control cells (5%). Treatment with rhamnetin (5 µΜ and 10 µΜ) decreased the apoptotic cell death induced by CRAB to 19% and 20%, respectively, as demonstrated in Figure 6C,D. Additionally, previous research has demonstrated that LPS exposure induces apoptosis in various cell types, including lung epithelial cells, endothelial cells, and RAW264.7 macrophages [16]. Therefore, we suggest the protective effects of rhamnetin against CRAB-induced apoptosis.

### 2.6. Rhamnetin Inhibits Apoptosis and Pyroptosis in CRAB-Stimulated Murine Macrophages

**Figure 7:**
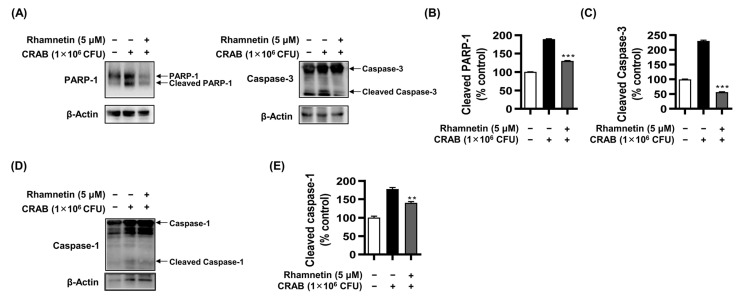
Rhamnetin inhibits apoptosis and pyroptosis in CRAB (1 × 10^6^ CFU)-stimulated murine macrophages. (**A**) Immunoblotting analysis showing decrease in levels of cleaved PARP-1 and caspase-3 in CRAB-stimulated cells with 5 µM rhamnetin. Bar graphs represent the percentage of (**B**) cleaved PARP-1 and (**C**) cleaved caspase-3 levels. (**D**) Decrease in levels of cleaved caspase-1 with 5 µM rhamnetin in CRAB-stimulated cells. β-actin was utilized as a loading control. Bar graphs represent the percentage of (**E**) cleaved caspase-1 levels. Results are shown as the mean ± SEM. ** *p* < 0.01 and *** *p* < 0.001 compared with the CRAB-stimulated group.

To confirm CRAB-induced apoptosis, we performed a western blot analysis, showing the effect of CRAB stimulation on the cleavage of PARP-1 and caspase-3 (Figure 7A–C). In CRAB-stimulated RAW264.7 cells, the expression of cleaved PARP-1 and caspase-3 was upregulated to 189% and 230%, respectively, compared to nonstimulated cells, while treatment with 5 μM rhamnetin decreased their expression significantly to 130% and 56%, respectively. These results suggested that rhamnetin protects macrophages against apoptosis induced by CRAB infection.

Given that LPS induces pyroptosis and necrosis in various cell lines, including macrophages [40,41], we proceeded to investigate whether CRAB infection also elicits pyroptosis in macrophages. The cleavage of caspase-1 stimulated by CRAB in macrophages was observed in an immunoblotting assay. The expression of cleavage of caspase-1 in CRAB-stimulated cells increased to 177% compared with that of noninfected cells. However, the treatment with 5 μM rhamnetin reduced the cleavage of caspase-1 to 140%, as shown in Figure 7D,E. These findings suggest that rhamnetin effectively protects macrophage cells against CRAB-induced pyroptosis.

We observed that rhamnetin has anti-inflammatory activity in a septic shock mouse model (Figure 2C), suppressing the serum levels of the inflammatory cytokines, such as IL-1β and IL-18, significantly. Based on these findings, we concluded that pyroptosis mediated by CRAB infection in mice is extensively inhibited by rhamnetin; this indicates a protective role of rhamnetin against CRAB-induced sepsis.

## 3. Discussion

In the present study, we demonstrated that rhamnetin protected murine macrophages against CRAB-induced apoptotic and pyroptotic cell death and inflammation. We evaluated the antioxidant activities in vitro using RAW264.7 macrophage cells and the antiseptic activities of rhamnetin in vivo using a CRAB-induced sepsis mouse model. Furthermore, we investigated the underlying mechanism of CRAB-induced macrophage cell death and septic shock.

Sepsis is a multifaceted syndrome in which an immune response to bacterial infections occurs abnormally, causing excessive inflammation and organ dysfunction [42]. In particular, infection by Gram-negative bacteria is an urgent problem because the outer cell membrane of bacteria consists of LPS, which not only causes aberrant cascades of the inflammatory response but also hinders the development of new antibiotics against MDR bacteria [43]. Antimicrobial therapy is the first line of sepsis/septic shock treatment. The early administration of empiric, antimicrobial therapy at the time of the sepsis’s identification and after the collection of the appropriate cultures is a crucial step in pharmacological management [44]. Patients with Gram-negative septic shock are treated with therapy such as carbapenem and colistin. However, the excessive use of these antibiotics has resulted in resistance to bacterial infection [2,7]. Therefore, the development of new antibiotics with low toxicity is necessary for the treatment of sepsis.

Owing to the lower toxicity of rhamnetin compared to other flavonoids, rhamnetin tends to scavenge free radicals and has high anti-inflammatory activity [31]. In this study, we investigated the potential of rhamnetin as a candidate for the treatment of CRAB-induced septic shock in mice and uncovered its cytoprotective role in CRAB-induced macrophage cell death. Therefore, we addressed the mechanistic role of rhamnetin in protecting mice from CRAB-induced septic shock and CRAB-induced apoptotic and/or pyroptotic cell death via the modulation of the ROS pathway. Our previous study demonstrated that rhamnetin inhibits the production of inflammatory cytokines murine tumor necrosis factor (mTNF)-α, macrophage inflammatory protein (mMIP)-1, and mMIP-2 via p38 mitogen-activated protein kinase (MAPK), extracellular signal-regulated kinase (ERK), and c-Jun N-terminal kinase (JNK) in LPS- or IFN-γ-stimulated RAW264.7 cells [34]. Furthermore, it showed antimycobacterial effects and suppressed the inflammatory cytokines interleukin (IL)-1β, IL-6, and IL-12, as well as matrix metalloproteinase-1, including TNF-α, through the inhibition of IFN-γ-mediated stimulation of ERK1 and p38 MAPK in human lung fibroblast MRC-5 cells, supporting its potent antituberculosis activity [45].

Individuals frequently develop chronic and acute inflammatory disorders. Bacterial endotoxins are the major causes of inflammatory diseases. LPS, the endotoxin released from the outer membranes of Gram-negative bacteria, stimulates TLR4, initiating intracellular signaling cascades that lead to the production of pro-inflammatory cytokines. These molecules are associated with immune responses and inflammation [46]. Therefore, new therapeutic molecules have been developed to prevent uncontrolled bacterial endotoxin-mediated sepsis by targeting the LPS-stimulated TLR4 signaling pathway to develop new antagonists by targeting TLR4 and MD-2 [47,48]. In this study, we investigated the regulatory role of rhamnetin in CRAB-induced sepsis. Rhamnetin neutralized the endotoxin in the lung lysates of CRAB-infected mice (Figure 2B), and CRAB-stimulated TLR4 activation was markedly reduced by rhamnetin, as evaluated by the SEAP assay in HEK-Blue^TM^ hTLR4 cells (Figure 3A). These results suggest that rhamnetin possesses strong anti-inflammatory potential, as demonstrated by modulating the TLR4-inflammatory signaling pathway in CRAB-stimulated HEK-Blue™ hTLR4 cells. This modulation may be achieved through the reduction of LPS released from CRAB-infected mice, leading to the suppression of pro-inflammatory cytokine production inhibition of the TLR4 signaling pathway. SPR analysis and docking simulation showed that rhamnetin strongly interacted with the hydrophobic pocket of MD-2, thereby inhibiting the binding of LPS to the TLR4/MD-2 complex and dimerization of the TLR4/MD-2 complex, followed by the inhibition of downstream signaling cascades of TLR4 (Figure 4A–D). Owing to its anti-inflammatory properties and binding to MD-2, rhamnetin can be used as a natural potential candidate for the treatment of TLR4-mediated inflammatory diseases.

Among the various forms of cell death, apoptosis is programmed and noninflammatory. Apoptosis in mammalian cells is induced by cysteine proteases [49,50], known as caspases. Apoptosis, stimulated by various extracellular or intracellular stimuli, is accompanied by the release of cytochrome c, specifically during mitochondria-mediated cell death. During apoptotic cell death, the cleavage of PARP-1 and caspase-3, 6, and 7 occurs [51,52]; this is followed by the activation of caspase-1, resulting in the conversion of pro-caspase-1 to its activated form [21,22]. In this type of cell death process, pro-inflammatory cytokines, including IL-1β and IL-18, are cleaved and subsequently released into the extracellular space through plasma membrane pores, thereby inducing inflammatory cell death [21]. Therefore, we investigated the role of rhamnetin in LPS-induced apoptosis and pyroptosis of macrophages, CRAB-induced sepsis in a mouse model, and CRAB-induced RAW264.7 cell death. Rhamnetin extensively inhibited CRAB- and LPS-stimulated apoptotic cell damage, as observed using flow cytometry (Figure 6A–D). In addition, we observed cleaved PARP-1 and caspase-3 in CRAB-stimulated macrophages using immunoblotting. Cleavage of PARP-1 and caspase-3 was reduced upon rhamnetin treatment (Figure 7A–C). Furthermore, the biochemical analysis showed that activation of the pyroptosis inducer caspase-1 was reduced by rhamnetin in CRAB-stimulated macrophages, showing a reduction in cleaved caspase-1 (Figure 7D,E), suggesting a cytoprotective role for rhamnetin against LPS- and/or CRAB-induced apoptotic and pyroptotic cell death.

ROS are extremely unstable and reactive free radicals. Uncontrolled acute inflammation caused by oxidative stress due to Gram-negative sepsis can result in cellular damage [16]. However, antioxidative enzymes, such as catalase and superoxide dismutase, formed during biochemical processes within host cells hinder the production of ROS following LPS stimulation [11]. It has been reported that various flavonoids eliminate ROS and enzymes that cause oxidation and, hence, have good antioxidant potential [53]. Rhamnetin was also found to be an effective antioxidant agent against K562 tumor cells [54] and to protect against miconazole-induced H9c2 cell death via the ROS pathway [55]. Another study has shown that rhamnetin acts as a strong inhibitor of the oxidative enzyme β-carotene-15,15′-dioxygenase in pig intestinal cells and inhibits oxidative stress due to ROS [56]. Consequently, we hypothesized that rhamnetin might regulate ROS production in macrophages and septic shock mice induced by CRAB, thereby exhibiting its protective effects through its antioxidant properties. Rhamnetin substantially reduced CRAB-induced ROS production (Figure 5A–D). The depletion of intracellular ROS further inhibited the activation of inflammasomes, followed by a decrease in pyroptosis through the suppression of caspase-1 cleavage. Additionally, treatment with rhamnetin downregulated the expression of cleaved PARP-1, caspase-3, and caspase-1 in CRAB-stimulated RAW264.7 macrophage cells (Figure 7A–E). As a result, we successfully demonstrated that rhamnetin protected macrophages against LPS/CRAB-induced cell injury and CRAB-induced sepsis in mice by modulating the ROS pathway, indicating that rhamnetin plays a crucial role in cytoprotection against CRAB-induced apoptosis. Importantly, the protective activity of rhamnetin was demonstrated through its shielding effect on TLR4 signaling. This study demonstrated the potential role of rhamnetin in protection against CRAB-induced sepsis. As the functional role of rhamnetin in the inhibition of apoptosis and pyroptosis via the ROS pathway is not clearly demonstrated here, further studies are required to elucidate this mechanism.

## 4. Materials and Methods

### 4.1. Bacteria Strains

We stimulated the murine macrophages and induced sepsis using CRAB NMS 1707, a clinically isolated carbapenem-resistant *A. baumannii* with the OXA-23 gene, which was obtained from the National Institute of Health Multidrug-Resistant Bacteria Specialized Pathogen Resources Bank (Osong, Republic of Korea).

### 4.2. Materials

Rhamnetin (purity ≥ 99% by HPLC) was purchased from the Indofine Chemical Company (Hillsborough, NJ, USA). Rhamnetin was dissolved in dimethyl sulfoxide (DMSO) at a concentration of 10 mg/mL. LPS extracted from *E. coli* O111:B4 was obtained from Sigma-Aldrich (St. Louis, MO, USA).

### 4.3. Animals

Institute of Cancer Research (ICR) mice (female, 6-week-old) were purchased from Orient Bio (Daejeon, Republic of Korea). As described previously [57], all ICR mice were housed under a pathogen-free and controlled-humidity and -temperature environment. All animal experiments were approved by the Institutional Animal Care and Use Committee (IACUC) of Konkuk University, Seoul, Korea (IACUC number: KU22174).

### 4.4. CRAB-Induced Sepsis Mouse Model

ICR mice of five groups (5 mice per group) were randomly divided. All mouse models of sepsis were intraperitoneally (i.p.) injected. The control group was injected with phosphate-buffered saline (PBS, pH = 7.4). The rhamnetin control group received only rhamnetin. An amount of 1 mg/kg of rhamnetin, based on our previous work, was used to examine its therapeutic potential [30,31,32]. For the bacterial control group, Mice were infected with 200 μL of CRAB (NMS 1707, 5 × 10^6^ CFU/mouse). The amount of 5 × 10^6^ CFU of CRAB was selected to be the dose that stimulates the cytokine production and increases the bacterial load in the organ sufficiently with a single injection but was chosen as the dose for animals to survive in 16 h. In the rhamnetin pretreatment group, 5 mice were injected with rhamnetin (1 mg/kg) 1 h prior to CRAB injection. After 16 h of injection, the mice were sacrificed by euthanasia, and the blood and vital organs, such as lung, liver, and kidney, were collected with 1000-fold ice-cold PBS for analysis. All organs were homogenized with PBS. For bacterial counts of organs, 10 μL of the homogenates were plated onto Luria-Bertani agar plates. Plates were incubated at 37 °C for 16 h. Endotoxin levels in the lung lysates were quantified using a Pierce LAL Chromogenic Endotoxin Quantitation Kit (Thermo Fisher Scientific, Waltham, MA, USA), as described previously [58]. The amount of inflammatory cytokines, such as TNF-α, IL-6, IL-1β, and IL-18, in the serum was measured using sandwich ELISA kits (R&D Systems, Minneapolis, MN, USA), as described previously [59].

### 4.5. Bacterial Culture and Infection

CRAB (NMS 1707) bacteria were grown from stock in 10 mL Luria–Bertani (LB) medium overnight at 37 °C with shaking at 220 rotations per minute (rpm). The next day, a subculture was performed until OD value at 600 nm became below 0.6. An amount of 1 mL subcultured bacteria was then boiled at 100 °C for 15 min and was centrifuged at 8000 rpm for 5 min. The supernatant was removed, and the bacterial pellet was mixed with 1 mL warmed-fresh medium. The macrophage cells were then infected with bacteria at (1 × 10^6^ CFU), and cells were incubated for 18 h at 30 °C. After 18 h, cells were then used for further experiments.

### 4.6. Secreted Embryonic Alkaline Phosphatase (SEAP) Assay

Blue™ hTLR4 cells were seeded in 96-well plates at 2.5 × 10^4^ per well, using HEK-Blue detection media (Invivogen, San Diego, CA, USA) and treated with rhamnetin (0–50 μM). After 1 h, cells were stimulated by CRAB (7.5 × 10^5^ CFU) for 16 h. After 16 h, SEAP production was determined based on the absorbance of the supernatant, measured at 620 nm with a SpectraMAX microplate reader (Molecular Devices, LLC, San Jose, CA, USA). The experiment was performed as described previously [60].

### 4.7. In Vitro Cytotoxicity Assay

HEK-293 cells were obtained from the Korea Cell Line Bank (Seoul, Republic of Korea). Blue™ hTLR4 cells and HEK-293 cells were cultured in DMEM (Welgene, Gyeongsan, Republic of Korea) supplemented with 10% FBS and 1% penicillin/streptomycin and incubated at 37 °C in a humidified 5% CO_2_ incubator. The cytotoxicity of rhamnetin against Blue™ hTLR4 cells and HEK-293 cells was measured using a WST-8 Cell viability Assay kit (Biomax Co., Guri, Republic of Korea). The absorbance was measured at 450 nm using a SpectraMAX microplate reader. The experiment was performed as described previously [61].

### 4.8. Surface Plasmon Resonance (SPR)

The binding affinity measurement for rhamnetin with MD-2 protein (R&D Systems, Minneapolis, MN, USA) was performed using SPR on a Biacore T200 Instrument (GE Healthcare, Chicago, IL, USA) at Korea Basic Science Institute, Seoul. The experiment was conducted as described previously [30]. In brief, 30 μg/mL of MD-2 protein was covalently immobilized to the sensor chip (Sensor Chip CM5; Cytiva, MA, USA) surface using standard EDC/NHS amine coupling method with sodium acetate buffer (pH 5.0) to a resonance value of 2500 [62]. Rhamnetin was dissolved in a running buffer composed of PBS (pH 8.0), 0.05% Tween 20, and 1% DMSO. The binding affinity of the rhamnetin to MD-2 protein was measured using a 1:1 binding assay of Biacore T200 Evaluation Software 3.0 (GE Healthcare).

### 4.9. Molecular Docking

The crystal structure of human MD-2 in complex with lipid IVa (2E59) as a receptor and the structure of rhamnetin, which were obtained from the PubChem compound database (PCID:5281691), were prepared for molecular docking simulation. AutoDock ViNA was implemented in YASARA software version 21.12.19 (YASARA Bioscience, Wien, Austria) [63]. For preparing the receptor, all residues were rigid except for the side chains of the hydrophobic residues (Tyr65, Phe76, Tyr102, Phe104, and Phe147). Rhamnetin was used as a ligand molecule, with all the backbone atoms kept flexible. A total of 100 docking runs were performed, and the best rhamnetin-MD-2 complex model was selected based on the binding energy, lowest dissociation constant, and the highest population of all clusters [64,65,66].

### 4.10. Measurement of Reactive Oxygen Species (ROS)

Intracellular ROS generation was evaluated using a DCFDA/H2DCFDA—Cellular ROS Assay Kit (ab113851; Abcam, Cambridge, UK). To determine the ROS levels upon rhamnetin treatment in CRAB-stimulated cells, 2′,7′-dichlorodihydrofluorescein diacetate (DCFDA, also known as H2DCFDA) staining was performed as described previously [67,68]. Briefly, the RAW264.7 cells (2 × 10^4^ cells/well) grown in 96-well plates were treated with or without rhamnetin for 1 h, and treated cells were then exposed to CRAB (2 × 10^4^ CFU) for 18 h. Cells were washed with 1× PBS and stained with 25 µM DCFDA solution for 45 min at 37 °C in the dark. Cells were washed to remove the excess DCFDA, and 100 µL 1× PBS was added to each well. The fluorescence intensity of DCF was measured either using a fluorescence microplate reader at Ex/Em = 485/535 nm in endpoint mode by using SpectraMax Gemini™ XPS/EM Microplate Readers (Molecular Devices, LLC, San Jose, CA, USA) or using a flow cytometer (BD FACSClibur; Becton, Dickinson and Company, Franklin Lakes, NJ, USA). ROS level was semiquantified, and the percentage of the ROS level was normalized with the control group.

### 4.11. Flow Cytometry Assay

Cell apoptosis and ROS production were analyzed using a flow cytometer. Briefly, RAW 264.7 cells (1 × 10^6^ cells/well) were grown in (60 × 15 mm) cell culture dishes. Cells at confluency were treated with or without rhamnetin for 1 h in a serum-free-condition medium, and treated cells were then exposed to CRAB (1 × 10^6^ CFU) for 18 h. Cells were counted and washed two times with ice-cold 1× PBS and suspended in Annexin V Binding Buffer. The cells were washed and stained with 5 μL of Annexin V FITC (eBioscience™ Annexin V-FITC Apoptosis Detection Kit; Thermo Fisher Scientific) in 100 µL cell suspension for 15 min in the dark at room temperature, and cells were again washed, and 5 μL of propidium iodide (PI) solution was added to 500 µL cell suspension in 1× Binding Buffer. Subsequently, the stained cells in cell suspension were analyzed using flow cytometry, and the number of apoptotic cells was evaluated. ROS analyses with the flow cytometer were performed as described in Section 4.10.

### 4.12. Western Blotting

Murine macrophage RAW264.7 cells were obtained from the Korean cell line bank and cultured as described previously [54]. RAW264.7 Cells were treated with or without rhamnetin for 1 h, and treated cells were then exposed to CRAB (1 × 10^6^ CFU) for 18 h. The cells were then harvested, washed with 1× cold PBS, and lysed with a radioimmunoprecipitation assay (RIPA) buffer containing freshly added Xpert protease and an Xpert phosphatase inhibitors cocktail solution (100×) (GenDEPOT, Barker, TX, USA). Immunoblotting was performed, taking the quantified protein extract as described previously [69], using primary antibodies, such as PARP-1 (#9542), Caspase-3 (#9662), and Caspase-1 (#24232), purchased from Cell Signaling Technology (Danvers, MA, USA), and β-actin (#sc-47778), purchased from Santa Cruz Biotechnology (Dallas, TX, USA).

### 4.13. Statistical Analysis

All experiments were performed at least in triplicate, and the data are presented as the mean ± standard error of the mean (SEM) of independent experiments. One-way and two-way analyses of variance (ANOVA) followed by Dunnett’s tests were performed using the GraphPad Prism software version 8.0.1 (GraphPad Software Inc., La Jolla, CA, USA). Values were considered statistically significant at * *p* < 0.05, ** *p* < 0.01, and *** *p* < 0.001; ns indicates not significant.

## 5. Conclusions

In conclusion, we demonstrated for the first time that rhamnetin promotes murine macrophage cell survival against LPS- and CRAB-induced oxidative stress and has antiseptic properties in CRAB-mediated sepsis by modulating the ROS pathway. Rhamnetin inhibited the production of pro-inflammatory cytokines (TNF-α, IL-6, IL-1β, and IL-18), thereby preventing pyroptosis and inflammation via the TLR4 signaling pathway. This effect was achieved by neutralizing LPS released during CRAB-induced septic shock in a mouse model. Furthermore, rhamnetin reduced LPS- or CRAB-stimulated macrophage cell death by decreasing the oxidative stress exerted by the accumulation of intracellular ROS, suggesting a new alternative mechanism for the antiseptic and cytoprotective roles of rhamnetin in septic mouse and macrophages. This study provides insights into the potential of rhamnetin as a therapeutic candidate for the treatment of sepsis.

## Data Availability

The data presented in this study are available on request from the corresponding author.
